# Using eye tracking to explore the relationship between autism traits, joint attention, cognition and adaptive functioning in preschool children

**DOI:** 10.1186/s11689-026-09714-z

**Published:** 2026-09-01

**Authors:** Anthea A Stylianakis, Hannah Pincham, Sparsh Chawla, Nisha E Mathew, Valsamma Eapen

**Affiliations:** 1 Discipline of Psychiatry and Mental Health, School of Clinical Medicine, University of New South Wales, Sydney, NSW Australia; 2 Academic Unit of Child and Adolescent Psychiatry, South Western Sydney Local Health District, Sydney, Australia; 3 Royal Brisbane and Women’s Hospital, Queensland Health, Brisbane, QLD Australia; 4 Discipline of Paediatrics and Child Health, School of Clinical Medicine, University of New South Wales, Sydney, NSW Australia

**Keywords:** Autism, Early Start Denver Model, Early intervention, Joint attention, Adaptive functioning, Cognitive development

## Abstract

There is significant heterogeneity in clinical presentations, brain-imaging findings, and genetic underpinnings of autism. Autism traits, particularly social affect and joint attention, and related domains of adaptive functioning and cognitive development, are key characteristics in determining autism “profiles”. This study aimed to understand such baseline profiles in preschool autistic children receiving early intervention (age range, 2–7 years old; sample size *N* = 67). We also examined the association between phenotypic pre-intervention characteristics and response to intervention using Early Start Denver Model (ESDM). Baseline characteristics prior to receiving early intervention demonstrated a link between eye tracking measure of joint attention, namely better gaze accuracy, and clinical profile of autism traits, particularly better social affect/social overtures and adaptive functioning. Further, better receptive and expressive language, visual reception and fine motor skills, and higher development quotients on a visual processing task, were found to be associated with more accurate eye movements on a joint attention task. Predictors of improvement in autistic traits post-intervention included higher age at entry to intervention and baseline developmental functioning. Specifically, social affect was found to change in the same direction as cognitive functioning post-ESDM intervention, especially with regard to visual reception, fine motor and receptive language skills. The findings also suggest that better social affect and joint attention, characterised by better social overtures and maintenance of attention, are predictive of more accurate eye gaze in a social exercise, thereby making these potential intervention targets in early intervention.

**Supplementary Information:**

The online version contains supplementary material available at https://doi.org/10.1186/s11689-026-09714-z.

## Background

Autism is a neurodevelopmental disorder that is characterised by differences in an individual’s social communication and restricted and repetitive interests and behaviours [1, 2]. Autism is a “spectrum” condition [3] and may be best understood as “autisms” with significant heterogeneity [4] in clinical presentations (i.e. social-communication function and restricted, repetitive patterns of behaviour and interests), cognition, language ability and adaptive functioning [5, 6]. There is also substantial evidence of biological heterogeneity, with significant variability in brain-imaging findings and genetic underpinnings [5, 7]. This broad spectrum of phenotypic, genotypic and neurological presentations of autism can raise challenges in terms of the field’s understanding of the disorder, as well as the determinants of intervention outcome.

Autistic children benefit from clinical interventions which are strengths-focused and maximise cognitive and functional gains [8]. Early intervention is well supported by evidence and maximises a child’s potential by taking advantage of the plasticity of the developing brain [9]. However, there is evidence of substantial heterogeneity in trajectories of cognitive and adaptive functioning in autistic children [10–14]. Consequently, it is challenging to identify what individual intervention is most appropriate for which child, and at which point in their development [15, 16]. For example, children who present with identical pre-intervention cognitive skills have been found to respond differently to interventions [17]. Differential treatment responses may be due to factors including cognitive functioning, verbal communication abilities, as well as autism trait severity (in particular imitation, social affect and play skills [18, 19]). For example, studies have found that baseline adaptive functioning and cognitive ability may contribute to better outcomes following early intervention [20], and the presence or absence of associated intellectual disability is among the most critical influences on outcomes in autism [21]. Furthermore higher cognitive ability at baseline has been found to predict a less severe autism diagnostic classification, and greater improvements in social-communication skills following engagement in interventions [22]. However, the precise factors that moderate response to treatment are not well understood [19, 23]. Given the benefits of effective early intervention for autistic children [20], it is crucial to further understand the impact of specific clinical characteristics on treatment outcomes, so that they can be better matched.

One category of intervention that is particularly geared towards the needs of young children are naturalistic developmental behavioural interventions, which use strategies that involve naturally occurring environments and activities. The Early Start Denver Model (ESDM) is a comprehensive, evidence-based Naturalistic Developmental Behavioural Intervention for autistic children aged 12 to 60 months. It merges Applied Behavior Analysis (ABA) with relationship-based approaches such as Pivotal Response Therapy (PRT) and developmental perspectives. ESDM focuses on play-based interaction using sensory-social routines to improve social-emotional, language, and cognitive skills. Key Features of ESDM include intervention occurring in a natural, familiar environment such as a home or daycare, and relationship-based interactions that focus on building positive, engaging, and enjoyable relationships to foster motivation and social interaction. ESDM is child-initiated and led, and teaching is embedded within the child’s interests in order to encourage communication and turn-taking. Individualised learning is designed for each child using a developmental curriculum of nearly 500 items. While studies report improved outcomes in autistic children who receive early interventions such as the ESDM (see [24] and [25] for meta-analysis and systematic review), there is significant variability in individual responses to treatment, with no single treatment found to benefit all autistic children [15, 26, 27].

The ESDM encourages reciprocal social interactions and shared engagement. Consequently, it is possible that children with an autism “profile” that is characterised by better social affect may show better outcomes following the ESDM when compared to those who demonstrate a “profile” characterised by lower social affect. Indeed, a study by Asta et al. [28] found that a more favourable response to ESDM was predicted by higher expressive and receptive language, communication ability and personal-social and emotional development, as well as less restricted and repetitive behaviours, interests or activities. Similarly, Eapen et al. [15] demonstrated that higher social affect and play skills predicted better ESDM outcomes in young children.

A key element of social affect which is critical in social and language development is joint attention [29]. Differences in joint attention are often present in autistic children and challenges in joint attention, often from the first year of life, are strongly predictive of a later autism diagnosis [30]. On the other hand, joint attention emerges between 9 and 12 months in non-autistic children and is typically consolidated by 18 months of age [31, 32]. Indeed, autistic preschoolers display impairments in both response to joint attention (RJA; characterised as the child’s ability to follow a line of gaze) and the initiation of joint attention (IJA; when the child directs another’s attention to a shared target) [33, 34]. Joint attention can be measured effectively in young children, regardless of their verbal and non-verbal functioning, using eye-tracking which can detect subtle shifts in visual attention [35]. Furthermore, joint attention is well established as a critical skill for social and cognitive development, and particularly language development. Interventions targeting joint attention and joint engagement skill development have resulted in considerable improvements in social communication in children with autism, even amongst minimally verbal children [9, 35–37]. Consequently, it is perhaps unsurprising that eye gaze, as a marker of joint attention, has been studied as part of understanding the diverse presentations that constitute autism [38].

## Aims

This study sought to understand how autistic traits, particularly differences in social affect, and joint attention, and related domains of adaptive functioning and cognitive development, can facilitate the creation of autism “profiles” and if such profiles can be used to predict treatment response in preschoolers receiving ESDM intervention. Aim one of this study was to better understand the link between these four phenotypic characteristics and the severity of autistic traits. We hypothesised that those with lower joint attention would present with lower cognitive development, lower adaptive functioning and more severe autistic traits, relative to those with higher joint attention. Aim two of this the study was to elucidate the association between clinical phenotypic and eye tracking pre-treatment characteristics (specifically autism traits, joint attention, adaptive functioning and cognitive development), and response to ESDM intervention. We hypothesised that joint attention and social affect levels pre-ESDM would predict a change in autistic traits, developmental outcomes and adaptive functioning post-ESDM. In particular, we hypothesised that those with better joint attention and social affect at baseline would benefit more from ESDM, relative to those with poorer joint attention levels and social affect.

In conclusion, there is significant heterogeneity in autism, and autistic children’s responses to early intervention. This study examined how certain autistic traits can facilitate the creation of autism “profiles” and the utility of such profiles predicting treatment response to ESDM.

## Method

### Participants

The current study was part of a larger nationwide research program [16] that focused on the predictors of early intervention in autism in a cohort of preschool children. Participants in the present study were from one of the sites in the larger study based in Sydney. Participants were aged 2–7 years old (youngest at Autism Diagnostic Observation Schedule Second Edition (ADOS-2) administration was 31.01 months of age, oldest was 72.17 months of age) and had a diagnosis of Autism Spectrum Disorder (*N* = 67) as defined in the DSM-5 [1], that was confirmed using the Autism Diagnostic Observation Schedule Second Edition (ADOS-2) [39]. There were no specific exclusion criteria applied to the participants, though no children had visual acuity problems.

Of the participants in the study, 41 (*n* = 35 male) received ESDM in a group model and 26 were in the Treatment as Usual group (*n* = 22 male).

The study was approved by the University of New South Wales Human Research Ethics Committee (HC14267) and The Sydney Children’s Hospital Network Human Research Ethics Committee (HREC/16/SCHN/47) and written informed consent was obtained from the participants’ parents or legal guardians. Participants were recruited for the study from 18.01.2016 to 08.04.2019.

### Clinical measures

A battery of assessments was completed consisting of assessments of autism traits, cognitive and developmental domains, and adaptive functioning at the baseline timepoint and at a second timepoint following a mean of 10 months (one academic year) of intervention.

### Autism traits

The Autism Diagnostic Observation Schedule-Second Edition [39] Module 1 was administered at baseline and follow-up (*n* = 49 at baseline). Raw scores from the assessment were converted into algorithm scores for Repetitive and Restricted Behaviour (RRB) and Social Affect domains, Total Score and Calibrated Severity Score (CSS was included so as to allow for comparisons across ages within our cohort [40]). Higher scores denote a higher level of autistic traits.

### Cognitive, language and developmental skills

Mullen Scales of Early Learning (MSEL) is a developmental assessment for children from birth to 68 months which produces age-equivalent scores in the domains of Visual Reception (VR), Fine Motor (FM), Receptive Language (RL), Expressive Language (EL) and Gross Motor [41]. The Gross Motor subscale was not administered for this study given the age of the children. For each domain, raw score and a corresponding age equivalence score was obtained. Standardized developmental quotients (DQs) were calculated for analysis, where the age equivalent score on each of the subscales was divided by the chronological age and then multiplied by 100 [42]. Higher scores are indicative of higher cognitive ability. All children who were assessed using MSEL were under 68 months of age at the time of administration.

### Adaptive functioning

The Vineland Adaptive Behaviour Scales-Second Edition (VABS-II) is a parent-report measure of a child’s adaptive functioning across the domains of communication (including expressive and receptive language), daily living skills, socialisation, and motor skills, with higher scores indicating higher levels of adaptive functioning [43].

### Eye tracking procedure and stimuli

Participants were seated approximately 60 cm in front of a 22” monitor with a video resolution of 1680 × 1050 pixels in a quiet room and shown novel stimuli for assessing joint attention (JA). The task was conducted at the recruitment site and took approximately five minutes. The Tobii X2-60 eye tracker was used, with eye tracking data analysed using Tobii Studio software (Tobii Technology AB, 2018). Eye gaze was tracked using corneal reflection via an infrared camera attached to the monitor, which provided time-stamped Cartesian coordinates of gaze position which were used to extract eye gaze measurements. Eye movements from both eyes were collected at a sampling rate of 60 recordings per second (60 Hz) with an accuracy of 0.5°. For accurate eye gaze tracking, built-in five-point calibration in Tobii studio was completed before administering the task.

The aim of the JA task was to assess the child’s ability to respond to JA behaviours by observing whether the child followed the actor’s gaze and how much they processed the object of mutual reference (target object). The JA paradigm incorporated eight videos (13s each) that featured a female actor with two different objects (toys; target and distractor) on a table in front of her.

Each video followed the same sequence: An attention getter (star) appeared on the actor’s face accompanied by sound for a duration of 3s, the actor looking directly at the camera and smiling for 3s to engage with the child (Phase one: direct gaze), the actor either (a) shifting and holding her eye gaze towards one of the toys (Eyes-Only condition) or (b) moving her head and shifting her eye gaze towards one of the toys (Head/Eyes condition) for 4 s (Phase two: gaze shift) and shifting gaze back at the camera for 2s. In each trial, the gazed-at-toy was considered the “target” and the other toy the “distracter”. The Tobii I-VT filter was used to classify the raw gaze coordinates into fixations. To avoid visual preservation from influencing the location of participants’ fixation, the position of the target (left/right) was counterbalanced. There were 8 trials in total. The presentation of the conditions was counterbalanced such that half the participants began the experiment with the Head/Eyes condition and the other half with the eyes-only condition. Please note that in eye-tracking studies the “attention getter” is placed on the face of the stimulus person to maximise social attention, however it was not a requirement that the participant look at the actor’s face in order for the trial to be included in the analysis. An illustration visually outlining the steps of the eye tracking procedure described above can be found in de Belen et al. (2023; [44]).

The criteria used to define a valid eye tracking trial was based on successful calibration, more than 50% of gaze fixations tracked during stimuli, and the observer rating the participant as attending to the trials. The observer was the researcher facilitating the eye tracking procedure, they ensured that the child was attending the task throughout the trials. Six successful trials (out of the eight in total) were required for a participant’s data to be included in the final analysis.

### Eye tracking measures

Two different eye tracking measures were adopted from previous studies [44].

Gaze accuracy (percentage of accurate gaze shifts) was computed by dividing the number of trials where the participant looked towards the same target as the actor (correct trials) by the total number of valid trials.

The standard difference score (SDS) was computed by subtracting the frequency with which the participant initially looked at the distractor object from the frequency with which the participant initially looked toward the target object.

### Intervention

#### Early start denver model

The Early Start Denver Model (ESDM) involves play-based, active engagement of the child across aspects of their development, with a focus on functional communication, social interaction, cognition, play, and positive behaviours. This study employed the previously published ESDM curriculum and teaching principles within a group setting [45]. Participants all received two half-hour intensive individualised ESDM therapy sessions per week (i.e. 1 h per week) and 15-to-20 h of ESDM group intervention. There were two rooms in the centre, with up to ten children in each room. The staff-to-child ratio was 1:4. The one-to-one sessions were conducted by a consistent key worker. While there was no specific parent training component to the intervention, optional parent education evenings were offered at the centre.

### Treatment as usual

Treatment as usual refers to the standard, community-based treatment accessed by participants, which generally include a mixture of speech therapy and/or occupational therapy depending on the needs, priorities, and funding available to families. The duration, frequency and delivery format of treatment as usual differed between participants depending on what services they were accessing in the community.

### Statistical analyses

Statistical analyses were conducted using IBM SPSS Statistics 27. Correlations between each eye-tracking measure and clinical measure were assessed using Spearman’s correlations. All analyses were subject to Bonferroni corrections to reduce the risk of Type 1 errors.

### Data analysis summary

#### Baseline measures

Eye tracking measures taken at baseline were correlated with autism traits, development and adaptive functioning as measured pre-intervention. Baseline analyses included all participants (i.e. those that went on to receive the ESDM intervention and those that went on to receive other community-based interventions).

#### Treatment outcomes

Eye gaze results taken at baseline were correlated with treatment response as measured by ADOS-2, MSEL and VABS scores following the ESDM intervention (i.e. outcomes were compared pre- and post-ESDM intervention). Baseline autism traits, development and adaptive functioning were compared to the difference in autism traits pre- and post-ESDM intervention. Finally, changes in ADOS outcomes pre- and post-ESDM intervention were compared to changes in MSEL and VABS pre- and post-ESDM intervention. For intervention outcomes, only those participants who received the ESDM intervention were included in the analyses as we were interested in the outcomes of the participants following the ESDM intervention in particular.

## Results

### Participant demographics at baseline

There was a total of 69 participants (male = 59) with an age range of 31.01 to 73.03 months, overall, mean age of 51.99 months and a standard deviation of 9.90 months. Forty-one (male = 35) participants were in the ESDM intervention condition (age range: 33.18 months to 63.47 months, mean age = 49.54 months, standard deviation = 7.69) and 28 (male = 24) were in the treatment as usual condition (age range: 31.01 months to 73.03 months, mean age of 55.48, standard deviation of 11.67).

The number of participants for each measure at baseline is detailed in Table 1.

**Table 1:** Number of participants for each measure analysed

	*N*
Gaze Accuracy	60
SDS	60
ADOS-2 (Module 1)	49
MSEL	65 (Expressive Language = 63)
Vineland	58 (Written language = 57)

Socioeconomic status was measured for 48 participants’ families based on their postcode of residence using the 2021 Socio-Economic Indexes for Areas (SEIFA) Index of Relative Socio-economic Disadvantage (IRSD). SEIFA is a product developed by the Australian Bureau of statistics that ranks areas in Australia according to relative socio-economic advantage and disadvantage [46]. Participants were mapped to Postal Areas (POA) and their scores were classified into quintiles (1 = most disadvantaged and 5 = least disadvantaged). In this sample, the quintile mode was 1 (most disadvantaged), and the quintile median was also 1 (most disadvantaged).

## Part 1: baseline measures

### Eye tracking and autism traits

#### Subscale analysis for eye tracking and ADOS

At baseline, there were negative correlations found between ADOS subscale scores and the two eye-tracking measures of joint attention, of which one, social affect and gaze accuracy, achieved statistical significance after correction for multiple comparisons (see Table 2), suggesting a link between better gaze accuracy and fewer autistic traits in the domain of Social Affect.

**Table 2:** Correlations between eye gaze data and baseline autism symptomatology

ADOS Subscale	Eye Tracking Variables	
	Gaze Accuracy	SDS
Social Affect	− 0.454(0.003)* **df = 38**	− 0.415(0.008) df = 38
Restricted Repetitive Behaviour	− 0.062(0.706) df = 38	− 0.119(0.465) df = 38
Total	− 0.373(0.018) df = 38	− 0.367(0.020) df = 38
Calibrated Severity Score-Total	− 0.169(0.298) df = 38	− 0.211(0.191) df = 38

*Denotes *p*-value significant following Bonferroni correction of 0.05/8 for ADOS subscales correlation

#### Item-level analysis for ADOS and social affect

In order to further understand the link between autistic traits and eye movements, an item-level analysis was undertaken for individual items in the Social Affect domain of the ADOS-2, given that this comparison was statistically robust. At the item level, a significant link between more accurate joint attention (gaze accuracy and SDS) and response to joint attention (B11, Module 1) and amount of social overture/maintenance of attention (B13a, Module 1) was found, see Table 3 for results. No other correlations were significant once multiple comparisons were corrected (largest r: item B14: *r*=-.368, *p*=.019, df = 38; See supporting information Tables 1 and 2).

**Table 3:** Correlations between ADOS social affect items and eye gaze variables

Social affect item	Eye Gaze Variables	
	Gaze Accuracy	SDS
B11	− 0.388(0.013) df = 38	− 0.513(< 0.001) df = 38*
B13a	− 0.521(< 0.001) df = 38*	− 0.478(0.002) df = 38

*Denotes *p*-value significant following Bonferroni correction of 0.05/32 for Social Affect “B” item-levels

### Eye tracking and cognitive development

#### Eye tracking and the MSEL

At baseline, there were significant positive correlations between eye-tracking variables and all developmental domains as measured in raw scores of MSEL at baseline (see Table 4), as well as Developmental Quotient (see Table 5). Specifically, more advanced receptive and expressive language, visual reception and fine motor skills were associated with more accurate eye movements during a joint attention task.

**Table 4:** Correlations between eye-tracking variables and developmental domains (MSEL Domain Raw Scores)

MSEL Domains	Eye Tracking Variables	
	Gaze Accuracy	SDS
Visual Reception	0.439(< 0.001)* **df = 55**	0.425(< 0.001)* **df = 55**
Fine Motor	0.445(< 0.001)* **df=0.55**	0.432(< 0.001)* **df = 55**
Receptive Language	0.515(< 0.001)* **df = 55**	0.504(< 0.001)* **df = 55**
Expressive Language	0.549(< 0.001)* **df = 54**	0.502(< 0.001)* **df = 54**

*Denotes *p*-value significant following Bonferroni correction of 0.05/8 for MSEL domains

**Table 5:** Correlations between eye-tracking variables and MSEL Domain Developmental Quotients

MSEL Domains	Eye Tracking Variables	
	Gaze Accuracy	SDS
Visual Reception DQ	0.391(0.003)*, **df = 55**	0.393(0.002)*, **df = 55**
Fine Motor DQ	0.424(0.001)*, **df = 55**	0.416(0.001)*, **df = 55**
Receptive Language DQ	0.506(< 0.001)* **df = 55**	0.491(< 0.001)* **df = 55**
Expressive Language DQ	0.555(< 0.001)* **df = 54**	0.492(< 0.001)* **df = 54**

*Denotes *p*-value significant following Bonferroni correction of 0.05/8 for MSEL domains

#### Eye tracking and verbal and non-verbal developmental quotient

At baseline, there were significant positive correlations between eye tracking measures and both MSEL verbal and nonverbal developmental quotients (as shown in Table 6). Higher development quotients were associated with greater joint attention.

**Table 6:** Correlations between MSEL VDQ and NVDQ, and Eye-Tracking Variables

DQ	Eye Tracking Variables	
	Gaze Accuracy	SDS
VDQ^#^	0.519(< 0.001)*, **df = 55**	0.475(< 0.001)*, **df = 55**
NVDQ^##^	0.414(0.001)*, **df = 55**	0.412(0.001)*, **df = 55**

*Denotes *p*-value significant following Bonferroni correction of 0.05/4 for MSEL domains

^#^VDQ=Verbal Developmental Quotient

^##^NVDQ = Non-Verbal Developmental Quotient

#### Eye tracking and adaptive functioning

At baseline, there were significant positive correlations between the eye-tracking variables (gaze accuracy and SDS) and receptive and expressive language, all domains of daily living skills, and socialisation, as measured using the VABS-II (see Table 7), suggestive of a link between better joint attention and better adaptive functioning capabilities in the aforementioned domains.

**Table 7:** Correlations between eye-tracking variables and domains of adaptive functioning

VABS-II Domains	Eye Tracking Variables	
	Gaze Accuracy	SDS
Communication		
Receptive Language	0.596(< 0.001)* **df = 48**	0.572(< 0.001)* **df = 48**
Expressive Language	0.556(< 0.001)* **df = 48**	0.487(< 0.001)* **df = 48**
Written Language	0.277(0.054) df = 47	0.272(0.059) df = 47
Daily living skills		
Personal	0.571(< 0.001)* **df = 48**	0.548(< 0.001)* **df = 48**
Domestic	0.537(< 0.001)* **df = 48**	0.497(< 0.001)* **df = 48**
Community	0.518(< 0.001)* **df = 48**	0.549(< 0.001)* **df = 48**
Socialisation		
Interpersonal Relationships	0.587(< 0.001)* **df = 48**	0.526(< 0.001)* **df = 48**
Play and Leisure	0.509(< 0.001)* **df = 48**	0.490(< 0.001)* **df = 48**
Coping Skills	0.488(< 0.001)* **df = 48**	0.452(0.001)* **df = 48**
Motor Skills		
Gross Motor	0.265(0.063) df = 48	0.226(0.114) df = 48
Fine Motor	0.375(0.007) df = 48	0.394(0.005) df = 48

*Denotes *p*-value significant following Bonferroni correction of 0.05/22 for VABS domains

## Part 2: post-ESDM measures

Given that joint attention is a significant predictor of autism traits, development and adaptive functioning, we sought to test whether eye tracking results on a joint attention task at baseline can be used to predict treatment response. In this component of the study, results were only taken from the 41 (*n* = 35 males) participants who received the ESDM intervention.

A comparison of participant response numbers for each measure in the ESDM group is reported in Table 8.

**Table 8:** Comparison of participant response numbers pre- and post-ESDM

	Pre-ESDM	Post-ESDM
ADOS-2	*N* = 38	*N* = 26
MSEL	*N* = 40	*N* = 29
Vineland	*N* = 35	*N* = 19

**Fig. 1:**
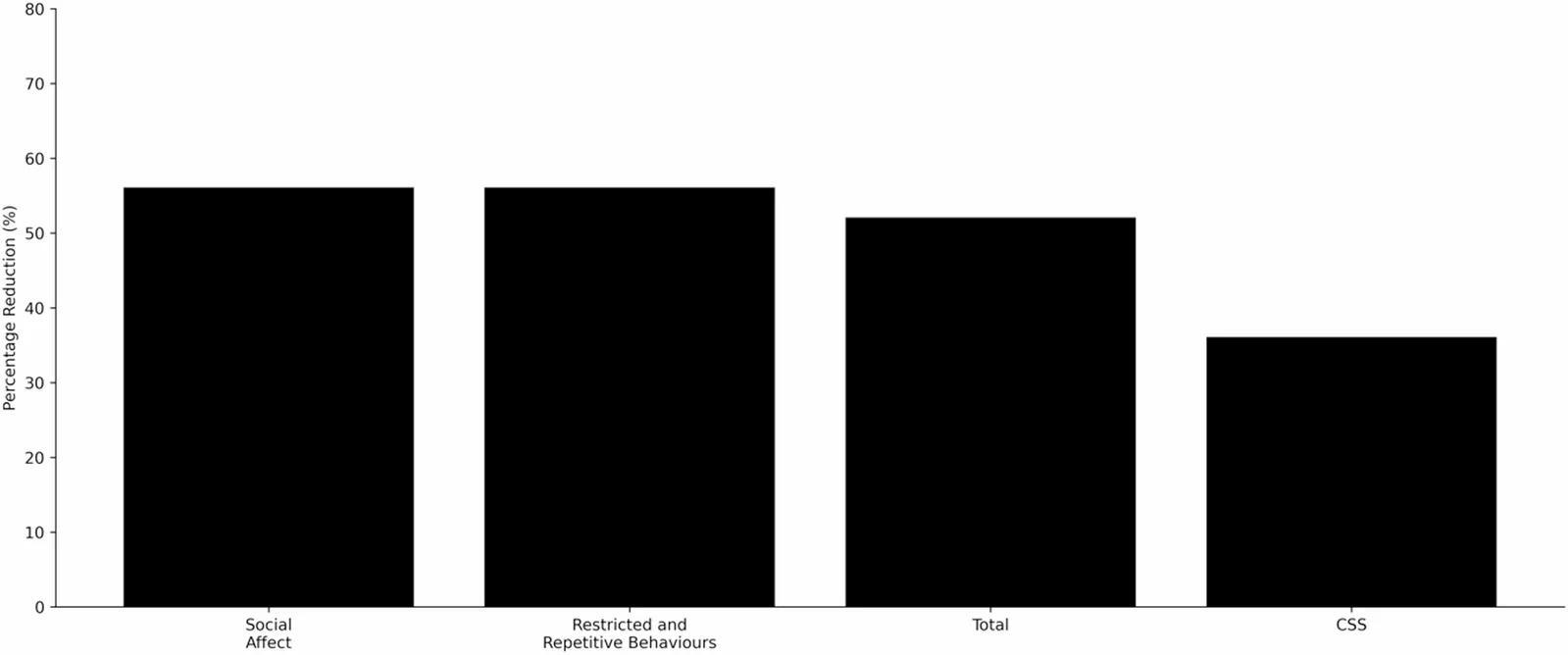
Percentage of participants whose autism symptoms reduced following ESDM intervention, as measured on ADOS domains (see supplementary Table 3 for details of number of children who had an increase or decrease in autism symptoms, or no change, post ESDM intervention)

**Fig. 2:**
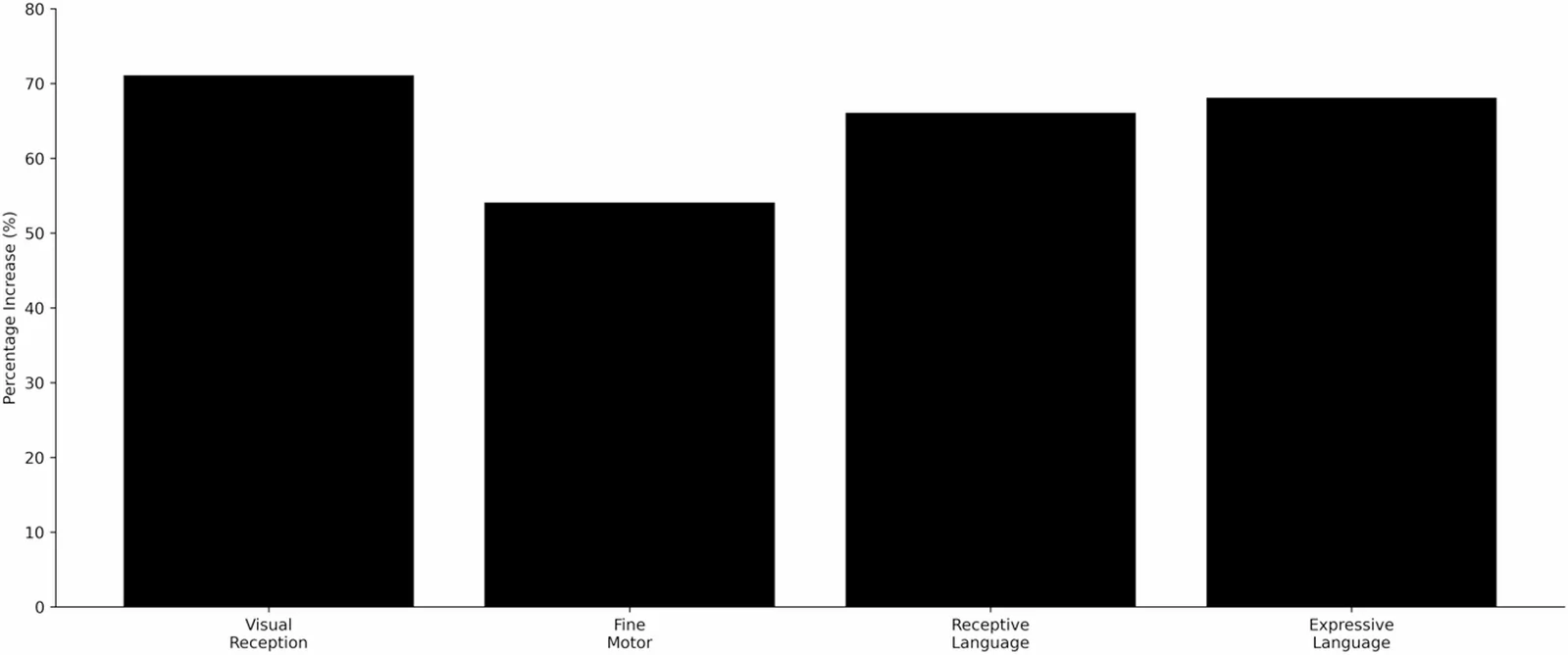
Percentage of participants whose cognitive functioning increased following ESDM intervention, as measured on MSEL (raw scores; see supporting information Table 4 for further details)

First, we analysed changes in autism traits, development and adaptive functioning post ESDM participation (Figs. 1 and 2, and Table 9).

**Table 9:** Percentage of participants whose adaptive functioning improved following ESDM intervention, as measured on Vineland domains (see supporting information Table 5 for further details)

Vineland domain (raw scores)	Percentage improvement in adaptive functioning following ESDM
Receptive Language	71%
Expressive Language	76%
Writing	50%
Personal	76%
Domestic	41%
Communication	53%
Relationships	65%
Play	59%
Coping	59%
Gross Motor	59%
Fine Motor	59%

### Eye tracking at baseline and changes post-ESDM intervention

There were no significant correlations between eye tracking results at baseline and intervention response scores across the ADOS-2 subscales following Bonferroni correction (i.e. the difference in scores pre- and post-treatment; largest r=-=0.471, *p*=.036, df = 18; SDS and Social Affect, Large Effect Size [47]; see supporting information Table 6 for results).

As with the baseline analysis, an item level analysis was conducted for the Social Affect subscale of the ADOS. There were no significant correlations between eye tracking results and pre-post-intervention scores at the item level for Social Affect following Bonferroni correction (largest r = A8 (gestures), SDS: *r*=-.564, *p*=.010, df = 18, Large Effect Size [47]; see supporting information Tables 7 and 8 for results).

There were also no significant correlations between eye tracking measures and intervention response scores (i.e. pre- and post-treatment difference scores) across the various domains of MSEL (Largest *r*=-.256, *p*=.227, df = 22, Expressive Language and Gaze Accuracy, Medium Effect Size [47]; see supporting information Table 9 for results).

Finally, there were no significant correlations between eye tracking measures and intervention response (i.e. pre- and post-treatment difference scores) for any of the Vineland domains (Largest *r* = − .311, *p*=.301, df = 11, SDS and Personal, Large Effect Size [47]; see supporting information Table 10 for results).

### Baseline autism symptomatology, cognition and development and changes in autism traits post-intervention

As eye tracking is a time-intensive measure that is not readily accessible to most clinicians, we sought to understand whether some of the additional information obtained using eye tracking correlated with measures that could be more easily administered and hence serve as a proxy for the deeper profile before a child receives an ESDM intervention. We also sought to understand how baseline functioning correlated to changes in autistic traits pre- and post-ESDM intervention. Changes in the ADOS-2 pre and post-ESDM were chosen as the intervention outcomes as the ADOS-2 is an assessment tool used to observe and evaluate behaviours related to autism directly.

#### Age at baseline and intervention response

There was an impact of age at baseline on changes in autism severity following ESDM, as measured by the CSS score on the ADOS-2, with a greater reduction in severity correlated with an increase in age at baseline (*r*=.591, *p*=.002, df = 22). There was no other significant impact of age at baseline on treatment response, as measured by changes in autism symptomatology (ADOS-2), development (MSEL) and adaptive functioning (Vineland) following Bonferroni correction (see supporting information Table 11). Furthermore, there was no significant age difference between participants who completed the MSEL, Vineland, and ADOS pre- and post-ESDM, and those who only completed these measures pre-ESDM (largest t = 1.44, df = 34, *p*=.160, Vineland).

#### Development at baseline and autism symptomatology outcomes on ADOS-2 domains

Developmental outcomes at baseline, as measured by scores on MSEL domains, were compared to the difference in autism symptomatology as measured by pre- and post-intervention difference scores on the ADOS-2 domains. Poorer visual reception scores on the MSEL at Baseline was a significant predictor of an improvement in the Social Affect domain of the ADOS post ESDM intervention following a Bonferroni correction (0.05/16; *r*=-.602, *p*=.002, df = 22; see supporting information Table 12 for detailed results).

In order to further understand the potential contributing factors to these results, developmental outcomes at baseline were compared to the pre- and post-intervention difference in autism symptomatology in the area of Social Affect on the ADOS-2. Following Bonferroni corrections no correlations reached statistical significance (largest *r*=-.576, *p*=.003, df = 22, Large Effect Size [47]; see supporting information Tables 13 and 14 for detailed results).

An improvement in autism traits pre-and post-intervention, particularly in the domains of Social Affect and total autism symptomatology, was significantly correlated with an improvement in development in the domains of the MSEL pre- and post-ESDM intervention following Bonferroni correction (0.05/16; see supporting information Table 15 for detailed results).

#### Adaptive functioning at baseline and autism symptomatology outcomes on ADOS-2 domains

Adaptive functioning at baseline, as measured by scores on the Vineland domains, were also compared to the difference in autism symptomatology as measured by pre- and post-intervention difference scores on the ADOS-2 domains. Following a Bonferroni correction there were no significant correlations between baseline adaptive functioning, as measured by scores on Vineland domains, and changes in autism symptomatology, as measured by changes in ADOS post ESDM intervention (largest *r*=-.568, *p*=.009, df = 18, Large Effect Size [47], ADOS Social Affect and Vineland Receptive Language; see supporting information Table 16 for detailed results).

Following Bonferroni corrections there were no significant correlations between Vineland domains at baseline and changes in ADOS Social Affect items post-ESDM treatment (largest *r*=.655, *p*=.002, df = 18, Large Effect Size [47], A1 and Receptive Language; see supporting information Tables 17 and 18 for detailed results).

An improvement in autism traits, as measured by a change in ADOS-2 domains pre-and post-intervention was not significantly correlated with an improvement in any domains of adaptive functioning as measured on the Vineland pre- and post-intervention following a Bonferroni correction (largest *r*=.636, *p*=.015, df = 13, Large Effect Size [47], Social Affect and Personal (Daily Living Skills Domain; see supplementary Table 19 for detailed results)). Again, after Bonferroni corrections were implemented, there were no significant correlations between changes in Vineland domains pre- and post-ESDM participation and ADOS Social Affect item changes pre- and post-ESDM participation (largest *r*= .670, *p*=.009, df = 12, Large Effect Size [47], A1 on the ADOS-2, Expressive Language; see supporting information Tables 20 and 21).

## Discussion

In summary, the first aim of this research was to understand the link between autistic traits, and particularly differences in social affect, joint attention, and related domains of adaptive functioning and cognitive development. Results revealed a significant link between eye tracking and social affect overall, as hypothesised. Findings also showed an association between eye tracking and the domains of response to joint attention and amount of social overture/maintenance of attention specifically. Furthermore, there was a significant positive association between eye tracking variables and cognitive development as well as measures of eye tracking and adaptive functioning, also as hypothesised. The second aim of the study was to understand the link between baseline clinical phenotypic characteristics in the areas of autism traits, joint attention, adaptive functioning and cognitive development and response to ESDM intervention. Contrary to our hypothesis we found no significant link between eye tracking results at baseline and ESDM intervention response scores across ADOS-2 subscales, in relation to cognitive development or adaptive functioning. We did however find an impact of age at baseline on changes in autism severity following ESDM.

The baseline data revealed a significant link between eye tracking measures and measures of autism traits, developmental profile and adaptive functioning, as was hypothesised. Specifically, there was a significant correlation between a child’s social affect and eye gaze accuracy. This suggests that higher levels of joint attention are associated with clinical measures of socio-communicative functioning, which is consistent with previous findings in a similar sample from the same larger nationwide research program [16, 44], and in line with the hypothesis of this study. Given that the domain of social affect on the ADOS was significantly linked to better levels of joint attention, and not the domain of restricted and repetitive behaviour, these results also point to an autism “profile” that is characterised by higher levels of social affect and joint attention. Social affect is a broad domain that encompasses many social, emotional, communication and behavioural facets. Consequently, it is important to understand exactly which aspects of social communication may be linked to measures of eye gaze in a social exercise to better understand phenotypic differences in autistic children, and how this may relate to their clinical presentation. To this end, a novel item level analysis in the current study showed that it was specifically those children with a higher quality and amount of social overtures and response to joint attention that performed more accurately on measures of eye gaze in a social exercise. These results reinforce the existing knowledge base on social communication in autistic children, which has shown a link between competency in joint attention in early childhood and the later development of social and language skills [48]. It is interesting to note that this item-level analysis found a link between eye gaze variables and aspects of the mechanics of coordinating attention (e.g. response to joint attention and social overture/maintenance of attention), but did not find a link between eye gaze variables and higher-level qualitative aspects of interaction (e.g. quality of social overtures, quality of social response and quality of rapport). This may be the consequence of quality of social interactions requiring the synchrony of multiple communicative behaviours including words, vocalisations, gestures as well as eye gaze. In contrast, eye gaze alone may reflect only one component of this system [49, 50].

In terms of the link between joint attention and development and adaptive functioning, children with better expressive and receptive language, as measured on both the MSEL and VABS, showed better joint attention. This adds further credence to previous findings which show a link between joint attention and the later development of language skills [48] and was also in line with our hypothesis. This would suggest the value of targeting joint attention in early intervention programs to improve core domains of social communication abilities in autism, with a focus on joint attention being a crucial aspect of ESDM, for example [51]. Further, the finding that a higher verbal and nonverbal development quotient on the MSEL correlated positively with measures of joint attention further supports a link between language and joint attention. This is also suggestive of early difficulties in joint social attention adversely affecting the development of language skills [52], given that early eye gaze behaviour is hypothesised to have a role in social language development [53]. Furthermore, the finding that a higher nonverbal development quotient on the MSEL correlated positive with measures of joint attention is in line with past research that demonstrates a relation between joint attention and non-verbal IQ [54].

The results of this study also found that those with better adaptive functioning in the areas of daily living skills and socialisation had better joint attention, as hypothesised. There are limited studies examining the link between joint attention and functional and adaptive outcomes directly [46]. However, previous research with a small sample of individuals has found a link between joint attention and only some aspects of adaptive functioning, such as socialisation but not communication or daily living skills [55]. Poon et al. [56] also found a significant link between joint attention and adaptive communication, however they did not assess socialisation or daily living skills. Finally, Harrison et al. [54] found that, in school-aged children, joint attention was significantly related to both verbal and non-verbal IQ and socialisation, communication and daily living domains of adaptive functioning. Nevertheless, the link between joint attention and socialisation (as measured in the Vineland) in this study may be explained by the role that joint attention plays in the development of social language and social behaviour [57]. The finding that higher joint attention abilities are associated with improved daily living skills is in line with the notion that impaired joint attention skills can adversely affect engagement in co-occupations, including daily living skills [58].

This study also found that, contrary to our initial hypothesis, joint attention levels at baseline did not predict a change in autistic traits, developmental outcomes, or adaptive functioning pre- and post-ESDM. These results may be in line with previous studies such as that by Bent et al. [59] which found that baseline social indicators such as joint attention may not be sufficient to predict outcomes after ESDM or EIBI. Further, while joint attention has been shown to predict language outcomes [57], sustained attention has emerged as a more important predictor of overall developmental outcomes than core autism features such as joint or social attention. This is possibly due to the fact that the child’s capacity to maintain attention may be a critical influencer of their ability to maximise learning opportunities [60].

We did however find that non-verbal cognitive ability at baseline was associated with significant improvements in social affect following ESDM intervention. These results add to a body of literature that demonstrate mixed results as to the impact of pre-intervention adaptive functioning and cognition on post-intervention outcomes on the ESDM or other similar interventions specifically (see [27] and [61] for review).

An improvement in autism symptomatology in the domain of social affect pre and post-intervention was found to be an important contributor to improvement in the developmental domains of visual reception, fine motor skills and receptive language post ESDM. Given the emphasis of the ESDM on sensory social routines, it is possible that children with improvements in social affect could utilise the program and benefit from it to a greater degree, resulting in greater gains in developmental domains. Conversely, an improvement in autism symptomatology in the domain of Restricted and Repetitive Behaviours pre- and post-intervention was only significantly correlated with an improvement in fine motor development. This finding is in keeping with the link between Restricted and Repetitive Behaviours and motor activities [62]. Further, an improvement in overall autism traits was not significantly correlated with an improvement in any domains of adaptive functioning. This finding is in line with previous research. Specifically, in a preschool cohort, Szatmari et al. [63] studied the developmental trajectories of autism trait severity and adaptive functioning in preschoolers, finding a low overlap between the severity of autistic traits and adaptive functioning in this cohort. Szatmari et al. [63] concluded that despite adaptive functioning challenges being a fundamental aspect of autism phenotypes, the developmental trajectories in autism and adaptive functioning are yoked to only a small degree. The results of the present study are important in adding to our current understanding of the impact of programs such as the ESDM on measures relating to autism. Specifically, these results suggest that changes in autism traits in the domain of social affect may move in the same direction as changes in cognitive functioning post ESDM participation. Conversely, changes in restricted repetitive behaviours may not move in the same direction as changes in cognitive functioning. Further, changes in adaptive functioning may also not move in the same direction as changes in autism symptomatology post-intervention. It should also be noted that the VABS, which was used to measure adaptive functioning in this study, is a parent-report measure, while MSEL and ADOS-2 are clinician administered. Therefore, differences in reported change may be influenced by contextual and reporter effects, with parent observations of everyday functioning in familiar contexts not necessarily mirroring clinician-observed changes in structured assessment settings.

Finally, the results of this study showed that the older the child was at the beginning of ESDM, the greater their reduction in autism severity post ESDM. Whilst this finding is not in keeping with some previous research, and with the hypothesis that, due to early sensitive periods in brain development, the earlier the intervention the better the response to the intervention [64, 65]). However, a recent meta-analysis involving 95 studies involving 4202 children in the intervention group, and 2000 controls found that age at intervention onset did not significantly predict the strength of post-intervention outcomes [61], which is consistent with our finding and suggest that other baseline characteristics may also be at play.

### Overall clinical implications

Interventions targeting joint attention and joint engagement skill development have resulted in considerable improvements in social communication in autistic children [26, 66, 67], even amongst minimally verbal children (relying primarily on gestures or single words to communicate [35]). However, prior studies using structured clinical measures of joint attention, such as the Early Social Communication Scales (ESCS) and Communication and Symbolic Behaviour Scales (CSBS) to predict later language ability [36, 68] and social competence [69, 70] have been limited due to the time and consumer burden posed by extensive assessments with long sitting time resulting in disengagement [71]. This is further compounded by the clinician skill needed to conduct the tests and inter-rater reliability issues [72]. Further, video-recording and later coding for the percentage of trials during which the child accurately responded to joint attention is required, and yet, this method is not optimal for precise determination of the child’s gaze [34]. Hence this paper adds to the growing interest in establishing a more objective measure of joint attention as a predictor of intervention response and longer-term outcomes. Eye tracking as a neurocognitive measure in autism research has gained momentum in recent years because it can provide more precise spatial and temporal information than face-to-face assessments by enhancing the detection of subtle shifts in gaze [73, 74]. In this regard, the results of this study suggest autistic traits, cognitive functioning and adaptive behaviours are linked to joint attention, which has clinical relevance in targeting interventions based on the child’s profile.

Eye tracking is relatively simple to use, non-invasive, and versatile for use in participants regardless of their verbal and non-verbal functioning, which makes it an ideal language-free tool. Unlike other measures, eye tracking does not require any equipment to be attached to the participant, which could otherwise be distressing or distracting to autistic children [75–77]. However, it should be noted that the child does need to sit still for long for calibration of the eye trackers, which may be challenging for some children. Other assessments of response to joint attention that are appropriate to a clinical context should also be considered for clinical utility, an example being the Dimensional Joint Attention Assessment (DJAA) by Elison et al. [78] which is a naturalistic play-based assessment of the emergence of RJA cue-reading abilities in the first two years of life. This assessment takes approximately 15 min to complete and measures RJA with high precision, often identifying early developmental risks.

### Limitations

The current study should be considered in light of its modest sample size, and wide age range relative to sample size. The participants in this sample were young children with high needs. Further, the assessments conducted in this study were comprehensive and labour intensive for both child (in the case of the ADOS) and parent (in the case of Mullens and Vineland). In light of the above, attrition in the number of participants pre- and post-intervention is to be expected. Unfortunately, this may have resulted in the study being underpowered, and the findings should be replicated with a larger sample size. Indeed, the large effect sizes of some of the non-significant pre-post intervention results may suggest that a larger sample size may allow for a more nuanced understanding of the impact of ESDM on autism symptomatology, cognitive development, and adaptive functioning.

Further, the study design is cross-sectional, such that causality between variables cannot be confirmed.

Another limiting factor is the high proportion of male participants. This is driven by ecological factors: autism is more commonly diagnosed in males than females [79].

### Future directions

Given that sex has been linked to phenotypic differences in the presentation of autism, it is important that future studies consider whether these findings can be replicated in pre-school aged girls on the autism spectrum.

Future research would also benefit from the use of eye tracking software options that allow online testing remotely as has been shown in our team’s research [80], which provides the opportunity to overcome time, transport, and geographic barriers in accessing eye tracking assessments by having to attend major facilities.

## Conclusions

This study found a link between social affect and levels of joint attention at baseline. Specifically, those children with a higher quality and amount of social overtures and maintenance of attention performed more accurately on measures of eye gaze in a social exercise. Furthermore, at baseline, children with better expressive and receptive language showed better joint attention, as did those with better adaptive functioning in the areas of daily living skills and socialisation. However, contrary to our initial hypothesis, joint attention levels at baseline did not predict a change in autistic traits, developmental outcomes, or adaptive functioning pre- and post-ESDM. We did however find that non-verbal cognitive ability at baseline was associated with significant improvements in social affect following ESDM intervention. An improvement in autism symptomatology in the domain of social affect pre and post-intervention was found to be an important contributor to improvement in the developmental domains of visual reception, fine motor skills and receptive language post ESDM. This paper adds to the field’s interest in establishing a more objective measure of joint attention as a predictor of intervention response and long-term outcomes.

## Supplementary Information


Supplementary Material 1.


## Data Availability

The datasets used and/or analysed during the current study are available from the corresponding author on reasonable request.
