# Supplementary material for: Using eye tracking to explore the relationship between autism traits, joint attention, cognition and adaptive functioning in preschool children
Source: J Neurodev Disord. 2026 Sep 1;18:49. doi: 10.1186/s11689-026-09714-z (PMC13531859; doi:10.1186/s11689-026-09714-z)
Supplement: Supplementary file 1 [file 11689_2026_9714_MOESM1_ESM.docx]

**Supporting information table 1**

*Correlations between ADOS social affect items A and eye gaze variables*

| Eye Gaze Variables | A1 | A2 | A3 | A4 | A5 | A6 | A7 | A8 |
| --- | --- | --- | --- | --- | --- | --- | --- | --- |
| Gaze Accuracy | -.307(.054) df=38 | .347(.028), df=38 | -.056(.748), df=33 | .274(.272), df=16 | .377(.123), df=16 | -.230(.154), df=38 | -.174(.284), df=38 | -.166(.307), df=38 |
| SDS | -.203(.210) df=38 | -.228(.157), df=38 | -.013(.939), df=33 | .387(.112), df=16 | .360(.143), df=16 | -.213(.188), df=38 | -.204(.206), df=38 | -.290(.070), df=38 |

**Supporting information table 2**

*Correlations between ADOS social affect items B and eye gaze variables*

| Eye Gaze Variables | B1 | B2 | B3 | B4 | B5 | B6 | B7 | B8 | B9 | B10 | B11 | B12 | B13a | B13b | B14 | B15 | B16 |
| --- | --- | --- | --- | --- | --- | --- | --- | --- | --- | --- | --- | --- | --- | --- | --- | --- | --- |
| Gaze Accuracy | - | -.194(.229), df=38 | -.320(.044), df=38 | -.255(.112), df=38 | -.241(.133), df=38 | -.295(.072), df=36 | .032(.844), df=37 | .204(.206), df=38 | -.202(.211), df=38 | -.095(.559), df=38 | -.388(.013), df=38 | .262(.103), df=38 | **-.521(p<.001), df=38** | -.532(.061), df=11 | -.368(.019), df=38 | -.166(.307), df=38 | -.115(.481), df=38 |
| SDS | - | -.065(.689), df=38 | -.259(.107), df=38 | -.103(.527), df=38 | -201(.213), df=38 | -.328(.044), df=36 | .089(.591), df=37 | .211(.192), df=38 | -.098(.547), df=38 | -.116(.475), df=38 | **-.513*(<.001), df=38** | -.272(.090), df=38 | .478*(.002), df=38 | -.490(.089), df=11 | -.284(.075), df=38 | -.127(.434), df=38 | -.155(.341), df=38 |

*Denotes p-value significant following Bonferroni correction of .05/64 for Social Affect “B” item-levels.

**Supporting information table 3**

*Number of children who had an increase or decrease in autism symptoms, or no change, post ESDM intervention.*

| ADOS Domains | No change | Increase in ASD symptoms | Decrease in ASD symptoms |
| --- | --- | --- | --- |
| Social Affect (n=25) | 3 | 8 | 14 |
| Restricted and Repetitive Behaviours (n=25) | 3 | 14 | 8 |
| Total (n=25) | 4 | 8 | 13 |
| CSS (n=25) | 7 | 9 | 9 |

**Supporting information table 4**

*Number of children who had an increase or decrease in cognitive functioning, or no change, post ESDM intervention.*

| Mullens Domains | No change | Increase in developmental domains | Decrease in developmental domains |
| --- | --- | --- | --- |
| VR (n=28) | 1 | 20 | 7 |
| FM (n=28) | 4 | 15 | 9 |
| RL (n=27) | 2 | 18 | 7 |
| EL (n=28) | 2 | 19 | 7 |
|  |  |  |  |

**Supporting information table 5**

*Number of children who had an increase or decrease in adaptive functioning, or no change, post ESDM intervention.*

| Vineland Domains | No change | Increase in adaptive behaviour | Decrease in adaptive behaviour |
| --- | --- | --- | --- |
| Receptive Language (n=17) | 1 | 12 | 4 |
| Expressive Language (n=17) | 0 | 13 | 4 |
| Wrt (n=18) | 3 | 9 | 6 |
| Personal (n=17) | 2 | 13 | 2 |
| Domestic (n=17) | 2 | 7 | 8 |
| Communication (n=17) | 2 | 9 | 6 |
| Relationships (n=17) | 0 | 11 | 6 |
| Play (n=17) | 2 | 10 | 5 |
| Coping (n=17) | 3 | 10 | 4 |
| Gross Motor (n=17) | 1 | 10 | 6 |
| Fine Motor (n=17) | 2 | 10 | 5 |

**Supporting information table 6**

*Correlation-Eye gaze baseline data and ADOS domains changes*

| Eye Gaze | Changes in ADOS | Social Affect | Restricted and repetitive behaviour | Total | CSS |
| --- | --- | --- | --- | --- | --- |
| Gaze Accuracy |  | -.424(.063) df=18 | .132(.578) df=18 | -.217(.358) df=18 | -.148(.533) df=18 |
| SDS |  | -.471(.036) df=18 | .068(.776) df=18 | -.281(.231) df=18 | -.206(.384) df=18 |

**Supporting information table 7**

*Correlation-Eye Gaze baseline and changes in ADOS Social Affect items A*

|  | Changes in ADOS | A1 | A2 | A3 | A4 | A5 | A6 | A7 | A8 |
| --- | --- | --- | --- | --- | --- | --- | --- | --- | --- |
| Eye Gaze Variables |  |  |  |  |  |  |  |  |  |
| Gaze Accuracy |  | .523(.018) df=18 | -.449(.047) df=18 | -.194(.456) df=15 | .312(.496) df=5 | .449(.312) df=5 | -.409(.073) df=18 | -.303(.194) df=18 | -.346(.136, df=18 |
| SDS |  | -.425(.062) df=18 | -.373(.105) df=18 | -.348(.171) df=15 | .417(.352) df=5 | .129(.783) df=5 | -.460(.041) df=18 | -.385(.093) df=18 | -.564(.010, df=18 |

**Supporting information table 8**

*Correlation-Eye Gaze baseline and changes in ADOS Social Affect items B*

|  | Change in ADOS | B1 | B2 | B3 | B4 | B5 | B6 | B7 | B8 | B9 | B10 | B11 | B12 | B13a | B14 | B15 | B16 |
| --- | --- | --- | --- | --- | --- | --- | --- | --- | --- | --- | --- | --- | --- | --- | --- | --- | --- |
| Eye Gaze Variables |  |  |  |  |  |  |  |  |  |  |  |  |  |  |  |  |  |
| Gaze Accuracy |  | - | -.124(.603) df=18 | .024(.919), df=18 | -.357(.122), df=18 | -.051(.829), df=18 | -.034(.897), df=15 | -.318(.185), df=17 | -.172(.469), df=18 | -.102(.668), df=18 | -.198(.404), df=18 | -.318(.172), df=18 | -.189(.425), df=18 | -.179(.450), df=18 | -.322(.166), df=18 | .159(.502), df=18 | .008(.973), df=18 |
| SDS |  | - | .300(.198), df=18 | .065(.785), df=18 | -.292(.211), df=18 | -.092(.699), df=18 | -.114(.664), df=15 | -.318(.184), df=17 | -.053(.824), df=18 | -.168(.480), df=18 | -.327(.159), df=18 | -.540(.014), df=18 | -.172(.468), df=18 | -.249(.290), df=18 | -.234(.320), df=18 | .061(.797), df=18 | -.130(.584), df=18 |

**Supporting information table 9**

*Correlation-Eye gaze baseline data and Mullens domains changes*

| Eye Gaze | Changes in Mullens | Visual Reception | Fine Motor | Receptive Language | Expressive Language |
| --- | --- | --- | --- | --- | --- |
| Gaze Accuracy |  | .000(1.000), df=22 | .072(.737), df=22 | -.047(.832), df=21 | -.256(.227), df=22 |
| SDS |  | .016(.942), df=22 | .194(.363), df=22 | .000(.999), df=21 | -.112(.603), df=22 |

**Supporting information table 10**

*Correlation-Eye gaze baseline data and Vineland domains changes*

|  | Changes in Vineland | Receptive Language | Expressive Language | Written | | Personal | | Domestic | Relationships | Comm | Play | Coping | Gross Motor | Fine Motor |
| --- | --- | --- | --- | --- | --- | --- | --- | --- | --- | --- | --- | --- | --- | --- |
| Eye Gaze Variables |  |  |  |  |  | |  | |  |  |  |  |  |  |
| Gaze Accuracy |  | -.134(.663), df=11 | -.070(.819), df=11 | .064(.827), df=12 | .294(.330), df=11 | | -.265(.381), df=11 | | -.118(.702), df=11 | .042(.891), df=11 | .038(.901), df=11 | .258(.394), df=11 | .144(.638), df=11 | -.057(.854), df=11 |
| SDS |  | -.236(.437), df=11 | -.038(.901), df=11 | .033(.911), df=12 | -.311(.301), df=11 | | -.200(.513), df=11 | | -.113(.712), df=11 | -.069(.822), df=11 | .080(.794), df=11 | .123(.688), df=11 | .110(.721), df=11 | -.085(.784), df=11 |

**Supporting information table 11**

*Correlation between changes in autism symptomatology (ADOS-2), development (Mullens) and adaptive functioning (Vineland) post ESDM intervention and age at baseline*

| Difference score pre- and post ESDM | Age at baseline |
| --- | --- |
| ADOS-Social Affect | .394(.057), df=22 |
| ADOS-Restricted and Repetitive behaviours | .354(.089), df=22 |
| ADOS-Total | .446(.029), df=22 |
| ADOS-Calibrated Severity Score | **.591*(.002), df=22** |
| Mullens-Visual Reception | -.041(.838), df=25 |
| Mullens Fine Motor | -.001(.996), df=25 |
|  |  |
| Mullens Receptive Language | .065(.754), df=24 |
| Mullens Expressive Language | -.056(.782), df=25 |
| Vineland Receptive Language | .277(.300), df=14 |
| Vineland Expressive Language | .354(.178), df=14 |
| Vineland Written | .085(.746), df=15 |
| Vineland Personal | .449(.081), df=14 |
| Vineland Domestic | .292(.273), df=14 |
| Vineland Gross Motor | .039(.885), df=14 |
| Vineland Fine Motor | .306(.248), df=14 |

**Supporting information table 12**

*Correlation between Mullens scores at baseline and ADOS-2 domain changes pre- and post-ESDM*

|  | Mullens Baseline | Visual Reception | Fine Motor | Receptive Language | Expressive Language |
| --- | --- | --- | --- | --- | --- |
| Change in ADOS |  |  |  |  |  |
| Social Affect |  | **-.602(.002)*, df=22** | -.462(.023), df=22 | -.474(.019), df=22 | -.234(.270), df=22 |
| Restricted and Repetitive Behaviour |  | -.207(.332), df=22 | -.281(.184), df=22 | -.015(.944), df=22 | -.043(.843), df=22 |
| Total |  | -.518(.009), df=22 | -.458(.025), df=22 | -.337(.108), df=22 | -.184(.390), df=22 |
| CSS |  | -.443(.030), df=22 | -.348(.096), df=22 | -.276(.191), df=22 | -.007(.973), df=22 |

*Denotes p-value significant following Bonferroni correction of .05/16

**Supporting information table 13**

*Correlation between Mullens scores at baseline and ADOS-2 Social Affect Item A changes pre- and post-ESDM*

|  | Changes in ADOS | A1 | A2 | A3 | A4 | A5 | A6 | A7 | A8 |
| --- | --- | --- | --- | --- | --- | --- | --- | --- | --- |
| Mullens Baseline |  |  |  |  |  |  |  |  |  |
| Visual Reception |  | -.489(.015), df=22 | -.490(.015), df=22 | .193(.401), df=19 | .224(.593), df=6 | .097(.819), df=6 | -.202(.343), df=22 | -.573(.003), df=22 | -.392(.058), df=22 |
| Fine Motor |  | -.576(.003), df=22 | -.537(.007), df=22 | -.099(.671), df=19 | .063(.883), df=6 | -.135(.749), df=6 | .058(.789), df=22 | -.400(.053), df=22 | -.129(.548), df=22 |
| Receptive Language |  | -.514(.010), df=22 | -.306(.145), df=22 | -.273(.232), df=19 | .473(.236), df=6 | -.126(.767), df=6 | -.382(.066), df=22 | -.421(.041), df=22 | -.402(.051), df=22 |
| Expressive Language |  | -.414(.044), df=22 | -.238(.262), df=22 | -.293(.198), df=19 | .552(.156), df=6 | -.288(.490), df=6 | -.248(.242), df=22 | -.292(.167), df=22 | -.336(.109), df=22 |

**Supporting information table 14**

*Correlation between Mullens scores at baseline and ADOS-2 Social Affect Item B changes pre- and post-ESDM*

|  | Changes in ADOS | B1 | B2 | B3 | B4 | B5 | B6 | B7 | B8 | B9 | B10 | B11 | B12 | B13a | B14 | B15 | B16 |
| --- | --- | --- | --- | --- | --- | --- | --- | --- | --- | --- | --- | --- | --- | --- | --- | --- | --- |
| Mullens Baseline |  |  |  |  |  |  |  |  |  |  |  |  |  |  |  |  |  |
| Visual Reception |  | - | .134(.532), df=22 | -.308(.143), df=22 | -.477(.018), df=22 | -.182(.394), df=22 | -.100(.666), df=19 | -.398(.060), df=21 | -.312(.137), df=22 | .021(.921), df=22 | -.110(.609), df=22 | -.501(.013), df=22 | -.296(.160), df=22 | -.179(.403), df=22 | -.504(.012), df=22 | -.019(.931), df=22 | -.022(.918), df=22 |
| Fine Motor |  | - | .029(.891), df=22 | -.121(.573), df=22 | -.353(.091), df=22 | -.117(.585), df=22 | .094(.685), df=19 | -.287(.184), df=21 | -.192(.368), df=22 | .010(.962), df=22 | -.039(.857), df=22 | -.270(.202), df=22 | -.135(.529), df=22 | -.062(.774), df=22 | -.306(.146), df=22 | .168(.434), df=22 | .154(.473), df=22 |
| Receptive Language |  | - | .176(.412), df=22 | -.031(.887), df=22 | -.436(.033), df=22 | -.311(.139), df=22 | -.165(.476), df=19 | -.274(.207), df=21 | -.177(.409), df=22 | .180(.400), df=22 | -.129(.548), df=22 | -.325(.121), df=22 | -.493(.014), df=22 | .010(.961), df=22 | -.308(.143), df=22 | -.161(.453), df=22 | -.062(.773), df=22 |
| Expressive Language |  | - | .078(.716), df=22 | .58(.787), df=22 | -.390(.060), df=22 | -.006(.978), df=22 | -.083(.721), df=19 | -.243(.263), df=21 | -.318(.131), df=22 | .235(.268), df=22 | .009(.968), df=22 | -.170(.427), df=22 | -.382(.065), df=22 | .098(.648), df=22 | -.257(.225), df=22 | -.077(.720), df=22 | .034(.876), df=22 |

**Supporting information table 15**

*Correlation between changes in Mullens scores pre- and post-ESDM and changes in ADOS-2 domains pre- and post-ESDM*

|  | Change in ADOS post ESDM intervention | | |  |  |  |
| --- | --- | --- | --- | --- | --- | --- |
| Change in Mullens post ESDM intervention |  |  |  | | |  |
|  | SA | RRB | Total | | | CSS |
| Visual Reception | **.605(.002)*, df=21** | .558(.006), df=21 | **.655(p<.001)*, df=21** | | | **.595(.003)*, df=21** |
| Fine Motor | **.614(.002)*, df=21** | **.654(p<.001)*, df=21** | **.704(p<.001)*, df=21** | | | **.637(.001)*, df=21** |
| Receptive Language | **.610(.002)*, df=21** | .455(.029), df=21 | **.610(.002)*, df=21** | | | .562(.005), df=21 |
| Expressive Language | .548(.007), df=21 | .553(.006), df=21 | **.615(.002)*, df=21** | | | .447(.032), df=21 |

*Denotes p-value significant following Bonferroni correction of .05/16

**Supporting information table 16**

*Correlation between changes in Vineland scores at baseline and changes in ADOS-2 domains pre- and post-ESDM*

|  | Vineland Baseline | Receptive Language | Expressive Language | Written | Personal | Domestic | Communication | Relationship | Play | Coping | Gross motor | Fine motor |
| --- | --- | --- | --- | --- | --- | --- | --- | --- | --- | --- | --- | --- |
| ADOS Difference |  |  |  |  |  |  |  |  |  |  |  |  |
| Social Affect |  | -.568(.009), df=18 | -.332(.153),df=18 | -.003(.991),df=19 | -.562(.010), df=18 | -.276(.239),df=18 | -.065(.784),df=18 | -.333(.152),df=18 | -.414(.070),df=18 | -.243(.302),df=18 | -.244(.301),df=18 | -.325(.162),df=18 |
| Restricted and Repetitive Behaviour |  | -.065(.785), df=18 | -.206(.383),df=18 | -.074(.748),df=19 | -.312(.180),df=18 | -.262(.264),df=18 | -.098(.682),df=18 | -.022(.926),df=18 | -.268(.253),df=18 | -.086(.718),df=18 | -.162(.495),df=18 | -.159(.502),df=18 |
| Total |  | -.425(.062), df=18 | -.329(.156),df=18 | -.038(.871),df=19 | -.540(.014),df=18 | -.317(.173),df=18 | -.092(.699),df=18 | -.241(.305),df=18 | -.416(.068),df=18 | -.210(.374),df=18 | -.247(.294),df=18 | -.302(.196),df=18 |
| CSS |  | -.211(.372), df=18 | -.160(.501),df=18 | .011(.961),df=19 | -.345(.137),df=18 | -.173(.467),df=18 | .013(.957),df=18 | -.143(.549),df=18 | -.280(.232),df=18 | -.098(.681),df=18 | -.255(.277),df=18 | .-.328(.158),df=18 |

**Supporting information table 17**

*Correlation between changes in Vineland scores at baseline and changes in ADOS-2 Social Affect Item A pre- and post-ESDM*

|  | Vineland Baseline | Receptive Language | Expressive Language | Written | Personal | Domestic | Communication | Relationship | Play | Coping | Gross motor | Fine motor |
| --- | --- | --- | --- | --- | --- | --- | --- | --- | --- | --- | --- | --- |
| ADOS Difference |  |  |  |  |  |  |  |  |  |  |  |  |
| A1 |  | -.655(.002), df=18 | -.618(.004), df=18 | -.106(.647), df=19 | -.565(.009), df=18 | -.378(.100), df=18 | -.378(.101), df=18 | -.563(.010), df=18 | -.638(.002), df=18 | -.484(.030), df=18 | -.194(.413), df=18 | -.114(.632), df=18 |
| A2 |  | -.261(.266), df=18 | -.215(.362), df=18 | -.142(.539), df=19 | -.205(.387), df=18 | -.089(.710), df=18 | -.095(.691), df=18 | -.123(.604), df=18 | -.286(.221), df=18 | -.143(.547), df=18 | -.114(.631), df=18 | -.184(.436), df=18 |
| A3 |  | -.046(.856),df=16 | -.034(.892), df=16 | .097(.693), df=17 | -.039(.879), df=16 | .075(.767), df=16 | .173(.492), df=16 | .072(.776),df=16 | r.103(.683),df=16 | -.024(.925),df=16 | .016(.949),df=16 | .203(.419),df=16 |
| A4 |  | .276(.549),df=15 | .457(.302),df=5 | r.218(.604),df=6 | .564(.187),df=5 | .611(.145),df=5 | .768(.044),df=5 | r.552(.198),df=5 | .692(.085),df=5 | -.081(.863),df=5 | .545(.206),df=5 | .317(.489),df=5 |
| A5 |  | -.137(.795),df=4 | -.623(.186),df=4 | -.130(.781),df=5 | -.322(.534),df=4 | -.246(.638),df=4 | -.464(.355),df=4 | -.309(.552),df=4 | r-.655(.158), df=4 | .223(.671),df=4 | -.308(.552),df=4 | -.036(.947),df=4 |
| A6 |  | -.334(.150),df=18 | -.084(.724),df=18 | -.062(.789),df=19 | -.367(.112),df=18 | -.144(.545),df=18 | -.044(.855),df=18 | -.332(.153),df=18 | -.087(.715),df=18 | -.254(.281),df=18 | .013(.958),df=18 | -.025(.917),df=18 |
| A7 |  | -.407(.075),df=18 | -.279(.233),df=18 | -.257(.261),df=19 | -.513(.021), df=18 | -.465(.039),df=18 | -.223(.344), df=18 | -.313(.179),df=18 | -.495(.027),df=18 | -.244(.300),df=18 | -.272(.246),df=18 | -.453(.045),df=18 |
| A8 |  | -.225(.340), df=18 | -.187(.430),df=18 | -.317(.162),df=19 | -.257(.274),df=18 | -.150(.527),df=18 | .057(.813),df=18 | -.237(.315),df=18 | -.295(.206),df=18 | -.148(.534), df=18 | -.026(.913), df=18 | .093(.698),df=18 |

**Supporting information table 18**

*Correlation between Vineland scores at baseline and changes in ADOS-2 Social Affect Item B pre- and post-ESDM*

|  | Vineland Baseline | Receptive Language | Expressive Language | Written | Personal | Domestic | Communication | Relationship | Play | Coping | Gross motor | Fine motor |
| --- | --- | --- | --- | --- | --- | --- | --- | --- | --- | --- | --- | --- |
| ADOS Difference |  |  |  |  |  |  |  |  |  |  |  |  |
| B1 | - |  | - | - | - | - | - | - | - | - | - | - |
| B2 |  | -.058(.809), df=18 | -.204(.387),df=18 | -.060(.795),df=19 | -.245(.297),df=18 | -.072(.763),df=18 | .080(.736),df=18 | .006(.980),df=18 | .060(.803),df=18 | -.023(.924),df=18 | .110(.645),df=18 | .209(.378),df=18 |
| B3 |  | .028(.908),df=18 | .351(.130),df=18 | -.215(.349),df=19 | -.166(.484),df=18 | -.194(.413),df=18 | ..049(.838),df=18 | -.141(.553),df=18 | .050(.835),df=18 | -.057(.811),df=18 | -.112(.638),df=18 | -.425(.062),df=18 |
| B4 |  | -.377(.102),df=18 | -.072(.764),df=18 | -.010(.964),df=19 | -.387(.092),df=18 | .-.402(.079),df=18 | -.211(.373),df=18 | -.047(.843),df=18 | .-.125(.600),df=18 | .108(.651),df=18 | -.046(.847),df=18 | -.514(.020),df=18 |
| B5 |  | -.520(.019),df=18 | -.364(.114),df=18 | .165(.476),df=19 | -.225(.340),df=18 | .004(.985),df=18 | .043(.856),df=18 | -.300(.199),df=18 | r-.259(.271),df=18 | -.249(.289),df=18 | -.073(.758),df=18 | .027(.909),df=18 |
| B6 |  | -.040(.875),df=16 | -.124(.624),df=16 | -.147(.547),df=17 | -.284(.254),df=16 | -.175(.487),df=16 | .044(.863),df=16 | .013(.960),df=16 | -.255(.307),df=16 | -.117(.645),df=16 | -.109(.667),df=16 | -.102(.687),df=16 |
| B7 |  | -.447(.055),df=17 | .080(.744),df=17 | -.044(.855),df=18 | -.316(.187),df=17 | -.116(.637),df=17 | .014(.955),df=17 | -.180(.461),df=17 | -.103(.675),df=17 | -.131(.593),df=17 | -.250(.301),df=17 | -.356(.135),df=17 |
| B8 |  | -.439(.053),df=18 | -.020(.935),df=18 | .000(1.000),df=19 | -.374(.104),df=18 | -.104(.662),df=18 | .044(.853),df=18 | -.213(.368),df=18 | -.078(.742),df=18 | -.124(.603),df=18 | -.250(.287),df=18 | -.135(.571),df=18 |
| B9 |  | .161(.499),df=18 | .069(.773),df=18 | .378(.091),df=19 | -.082(.732),df=18 | .005(.984),df=18 | .206(.384),df=18 | .264(.260),df=18 | .074(.756),df=18 | .276(.240),df=18 | .141(.553),df=18 | .035(.883),df=18 |
| B10 |  | -.253(.282),df=18 | -.435(.055),df=18 | .185(.423),df=19 | -.366(.112),df=18 | -.066(.783),df=18 | -.012(.959),df=18 | -.158(.505),df=18 | -.291(.213),df=18 | -.024(.918),df=18 | -.037(.878),df=18 | .167(.482),df=18 |
| B11 |  | -.356(.123),df=18 | -.114(.632),df=18 | -.143(.537),df=19 | -.424(.062),df=18 | -.437(.054),df=18 | -.225(.340),df=18 | -.308(.186),df=18 | -.550(.012),n=20 | -.180(.448),df=18 | -.378(.100),df=18 | -.482(.031),df=18 |
| B12 |  | -.654(.002),df=18 | -.536(.015),df=18 | -.187(.417),df=19 | -.559(.010),df=18 | -.249(.289),df=18 | -.133(.577),df=18 | -.395(.084),df=18 | -.274(.243)df=18 | -.422(.064),df=18 | .089(.710),df=18 | .067(.779),df=18 |
| B13a |  | -.232(.325),df=18 | -.341(.141),df=18 | .121(.601),df=19 | -.247(.293),df=18 | -.118(.622),df=18 | -.082(.731),df=18 | -.084(.726),df=18 | -.355(.124),df=18 | .072(.764),df=18 | .207(.382),df=18 | .212(.369),df=18 |
| B14 |  | -.333(.151),df=18 | -.110(.644),df=18 | -.110(.635),df=19 | -.456(.043),df=18 | -.380(.098),df=18 | -.005(.984),df=18 | -.221(.349),df=18 | -.376(.102),df=18 | -.042(.861),df=18 | -.147(.537),df=18 | -.241(.306),df=18 |
| B15 |  | -.155(.513),df=18 | -.151(.526),df=18 | .045(.845),df=19 | -.537(.015),df=18 | -.156(.510),df=18 | .121(.610),df=18 | -.003(.988),df=18 | -.124(.604),df=18 | .101(.673),df=18 | -.074(.756),df=18 | -.129(.587),df=18 |
| B16 |  | -.068(.776),df=18 | -.102(.669),df=18 | .158(.494),df=19 | -.395(.085),df=18 | -.030(.901),df=18 | .209(.377),df=18 | .043(.857),df=18 | -.102(.670),df=18 | .117(.624),df=18 | -.027(.909),df=18 | .008(.973), df=18 |

**Supporting information table 19**

*Correlation between changes in Vineland scores pre- and post-ESDM and changes in ADOS-2 domains pre- and post-ESDM*

|  | Vineland Difference | Receptive Language | Expressive Language | Written | Personal | Domestic | Communication | Relationship | Play | Coping | Gross motor | Fine motor |
| --- | --- | --- | --- | --- | --- | --- | --- | --- | --- | --- | --- | --- |
| ADOS Difference |  |  |  |  |  |  |  |  |  |  |  |  |
| Social Affect |  | .601(.023), df=12 | .467(.092), df=12 | .217(.437), df=13 | .636(.015), df=12 | .357(.210), df=12 | .464(.094), df=12 | .502(.067), df=12 | .306(.287), df=12 | .320(.265), df=12 | -.176(.546), df=12 | -.030(.918), df=12 |
| RRB |  | .256(.376), df=12 | .419(.136), df=12 | -.146(.604), df=13 | .591(.026), df=12 | .060(.839), df=12 | .419(.136), df=12 | .547(.043), df=12 | .504(.066), df=12 | .102(.728), df=12 | -.430(.125), df=12 | -.061(.836), df=12 |
| Total |  | .548(.042), df=12 | .532(.050), df=12 | .085(.763), df=13 | .735(.003), df=12 | .281(.331), df=12 | .531(.051), df=12 | .619(.018), df=12 | .460(.098), df=12 | .275(.341), df=12 | -.333(.245), df=12 | -.051(.863), df=12 |
| CSS |  | .402(.154), df=12 | .361(.204), df=12 | -.207(.459), df=13 | .579(.030), df=12 | .092(.755), df=12 | .436(.119), df=12 | .342(.232), df=12 | .384(.176), df=12 | .274(.343), df=12 | -.355(.213), df=12 | -.011(.971), df=12 |

**Supporting information table 20**

*Correlation between changes in Vineland scores pre- and post-ESDM and changes in ADOS-2 Social Affect Item A pre- and post-ESDM*

|  | Vineland Difference | Receptive Language | Expressive Language | Written | Personal | Domestic | Communication | Relationship | Play | Coping | Gross motor | Fine motor |
| --- | --- | --- | --- | --- | --- | --- | --- | --- | --- | --- | --- | --- |
| ADOS Difference |  |  |  |  |  |  |  |  |  |  |  |  |
| A1 |  | .656(.011), df=12 | .670(.009), df=12 | .568(.027), df=13 | .583(.029), df=12 | .370(.193), df=12 | .390(.168), df=12 | .762(.002), df=12 | .387(.171), df=12 | .280(.333), df=12 | -.073(.805), df=12 | -.090(.760), df=12 |
| A2 |  | .337(.238), df=12 | .226(.437), df=12 | .395(.145), df=13 | .402(.154), df=12 | .261(.368), df=12 | .298(.302), df=12 | .318(.268), df=12 | .119(.685), df=12 | .187(.521), df=12 | -.038(.897), df=12 | -.045(.879), df=12 |
| A3 |  | .068(.826), df=11 | .127(.680), df=11 | .099(.735), df=12 | .314(.296), df=11 | -.104(.735), df=11 | .144(.640), df=11 | .152(.619), df=11 | -.016(.960), df=11 | .082(.789), df=11 | -.246(.418), df=11 | -.200(.513), df=11 |
| A4 |  | -.347(.653), df=2 | -.468(.532), df=2 | -.881(.048), df=3 | .460(.540), df=2 | -.465(.535), df=2 | -.311(.689), df=2 | -.911(.089), df=2 | .286(.714), df=2 | .879(.121),df=2 | .598(.402), df=2 | -.566(.434), df=2 |
| A5 |  | -.143(.909), df=1 | .062(.961), df=1 | -.098(.902), df=2 | .118(.925), df=1 | -.106(.932), df=1 | -.143(.909), df=1 | .893(.297), df=1 | -.266(.828), df=1 | -.980(.128), df=1 | -.991(.088), df=1 | .419(.725), df=1 |
| A6 |  | .481(.082), df=12 | .373(.189), df=12 | .531(.042), df=13 | .414(.141), df=12 | .457(.100), df=12 | .204(.483), df=12 | .156(.594), df=12 | .012(.967), df=12 | .447(.109), df=12 | -.084(.776), df=12 | -.382(.178), df=12 |
| A7 |  | r=.640, p=.014, df=12 | .526(.053), df=12 | .207(.460), df=13 | .771(.001), df=12 | .516(.059), df=12 | .645(.013), df=12 | .495(.072), df=12 | .430(.125), df=12 | .264(.361), df=12 | -.123(.676), df=12 | .053(.858), df=12 |
| A8 |  | r=.344, p=.229, df=12 | .141(.631), df=12 | .155(.581), df=13 | .412(.143), df=12 | .182(.534), df=12 | .073(.804), df=12 | .056(.850), df=12 | .051(.861), df=12 | .365(.199), df=12 | -.121(.680), df=12 | -.142(.629), df=12 |

**Supporting information table 21**

*Correlation between changes in Vineland scores pre- and post-ESDM and changes in ADOS-2 Social Affect Item B pre- and post-ESDM*

|  | Vineland Difference | Receptive Language | Expressive Language | Written | Personal | Domestic | Communication | Relationship | Play | Coping | Gross motor | Fine motor |
| --- | --- | --- | --- | --- | --- | --- | --- | --- | --- | --- | --- | --- |
| ADOS Difference |  |  |  |  |  |  |  |  |  |  |  |  |
| B1 | - | - | - | - | - | - | - | - | - | - | - | - |
| B2 |  | .359(.208), df=12 | .411(.144), df=12 | .276(.319), df=13 | .560(.037), df=12 | .323(.259), df=12 | .018(.950), df=12 | .615(.019), df=12 | .392(.165), df=12 | .377(.184), df=12 | -.341(.233), df=12 | -.508(.064), df=12 |
| B3 |  | -.122(.677), df=12 | -.276(.339), df=12 | -.352(.199), df=13 | -.023(.939), df=12 | .033(.910), df=12 | .111(.705), df=12 | -.294(.308), df=12 | -.012(.968), df=12 | .129(.660),df=12 | -.125(.671), df=12 | .041(.888), df=12 |
| B4 |  | .243(.402), df=12 | .154(.598), df=12 | .333(.225), df=13 | .343(.230), df=12 | .340(.234), df=12 | .521(.056), df=12 | .153(.602), df=12 | -.055(.851), df=12 | -.306(.287), df=12 | -.139(.637), df=12 | .243(.403), df=12 |
| B5 |  | .115(.695), df=12 | .072(.806),df=12 | .243(.383), df=13 | .052(.859), df=12 | .042(.885), df=12 | -.146(.620), df=12 | .271(.349), df=12 | -.126(.667), df=12 | .011(.970), df=12 | -.127(.666), df=12 | -.204(.484), df=12 |
| B6 |  | .550(.051), df=11 | .398(.179), df=11 | .416(.139), df=12 | .501(.081), df=11 | .474(.102), df=11 | .048(.875), df=11 | .182(.552), df=11 | .206(.500), df=12 | .479(.097), df=11 | -.120(.696), df=11 | -.262(.387), df=11 |
| B7 |  | .004(.990), df=11 | -.226(.458), df=11 | .309(.282), df=12 | -.118(.700), df=11 | .114(.710), df=11 | .008(.979), df=11 | -.238(.433), df=11 | -.405(.170), df=12 | -.074(.810), df=11 | .216(.479), df=11 | .159(.605), df=11 |
| B8 |  | .158(.590), df=12 | -.007(.982), df=12 | .055(.847), df=12 | .173(.555), df=12 | .160(.585), df=12 | .184(.530), df=12 | .050(.865), df=12 | -.101(.731), df=12 | .111(.705), df=12 | .071(.808), df=12 | .042(.886), df=12 |
| B9 |  | .487(.078), df=12 | .555(.039), df=12 | -.265(.340), df=13 | .599(.024), df=12 | .208(.475), df=12 | .498(.070), df=13 | .564(.036), df=12 | .611(.020), df=12 | .254(.381), df=12 | -.314(.274), df=12 | .067(.821), df=12 |
| B10 |  | .447(.109), df=12 | .594(.025), df=12 | -.032(.909), df=13 | .505(.066), df=12 | .083(.777), df=12 | .217(.456), df=12 | .556(.039), df=12 | .349(.222), df=12 | .102(.728), df=12 | -.457(.100), df=12 | -.161(.583), df=12 |
| B11 |  | .609(.021), df=12 | .335(.242), df=12 | .244(.381(, df=13 | .414(.141), df=12 | .585(.028), df=12 | .478(.083), df=12 | .217(.456), df=12 | .295(.306), df=12 | .195(.504), df=12 | .299(.299), df=12 | .401(.155), df=12 |
| B12 |  | .555(.039), df=12 | .528(.052), df=12 | .493(.062), df=13 | .530(.051), df=12 | .405(.151), df=12 | .155(.597), df=12 | .443(.112), df=12 | .128(.663), df=12 | .422(.133), df=12 | -.433(.122), df=12 | -.563(.036), df=12 |
| B13a |  | .650(.012), df=12 | .560(.037), df=12 | -.095(.736), df=13 | .505(.065), df=12 | .388(.170), df=12 | .443(.112), df=12 | .514(.060), df=12 | .611(.020), df=12 | .383(.176), df=12 | -.091(.756), df=12 | .185(.527), df=12 |
| B14 |  | .501(.068), df=12 | .343(.229), df=12 | .468(.079), df=13 | .554(.040), df=12 | .625(.017), df=12 | .252(.384), df=12 | .410(.145), df=12 | .134(.648), df=12 | .084(.775), df=12 | -.248(.393), df=12 | -.104(.723), df=12 |
| B15 |  | .357(.210), df=12 | .366(.198), df=12 | .030(.917), df=13 | .446(.110), df=12 | .237(.416), df=12 | .095(.748), df=12 | .159(.588), df=12 | .207(.477), df=12 | .235(.418), df=12 | -.428(.127), df=12 | -.217(.456), df=12 |
| B16 |  | .374(.188), df=12 | .407(.148), df=12 | .048(.866), df=13 | .616(.019), df=12 | .247(.395), df=12 | .179(.539), df=12 | .361(.205), df=12 | .211(.469), df=12 | .251(.387), df=12 | -.595(.025), df=12 | -.377(.183), df=12 |
